# Lethal exposure: An integrated approach to pathogen transmission via environmental reservoirs

**DOI:** 10.1038/srep27311

**Published:** 2016-06-06

**Authors:** Wendy C. Turner, Kyrre L. Kausrud, Wolfgang Beyer, W. Ryan Easterday, Zoë R. Barandongo, Elisabeth Blaschke, Claudine C. Cloete, Judith Lazak, Matthew N. Van Ert, Holly H. Ganz, Peter C. B. Turnbull, Nils Chr. Stenseth, Wayne M. Getz

**Affiliations:** 1 Centre for Ecological and Evolutionary Synthesis (CEES), Department of Biosciences, University of Oslo, P.O. Box 1066 Blindern, 0361 Oslo, Norway; 2 Department of Biological Sciences, State University of New York, Albany, New York 12222, USA; 3 Department of Environmental Science, Policy and Management, University of California, Berkeley, 137 Mulford Hall, Berkeley, CA 94720-3112, USA; 4 Institute of Animal Sciences, Department of Environmental and Animal Hygiene, University of Hohenheim, Hohenheim, Germany; 5 Department of Biological Sciences, Faculty of Science, University of Namibia, Windhoek, Namibia; 6 Etosha Ecological Institute, Ministry of Environment and Tourism, Etosha National Park, PO Box 6, Okaukuejo, Namibia; 7 Institute of International Animal Health, Free University of Berlin, Königsweg 67, 14163 Berlin, Germany; 8 Emerging Pathogens Institute, University of Florida, Gainesville, FL, USA; 9 Genome Center and Department of Evolution and Ecology, University of California, Davis, CA, USA; 10 Retired, Salisbury, UK; 11 School of Mathematical Sciences, University of KwaZulu-Natal, Private Bag X54001, Durban 4000, South Africa

## Abstract

To mitigate the effects of zoonotic diseases on human and animal populations, it is critical to understand what factors alter transmission dynamics. Here we assess the risk of exposure to lethal concentrations of the anthrax bacterium, *Bacillus anthracis*, for grazing animals in a natural system over time through different transmission mechanisms. We follow pathogen concentrations at anthrax carcass sites and waterholes for five years and estimate infection risk as a function of grass, soil or water intake, age of carcass sites, and the exposure required for a lethal infection. Grazing, not drinking, seems the dominant transmission route, and transmission is more probable from grazing at carcass sites 1–2 years of age. Unlike most studies of virulent pathogens that are conducted under controlled conditions for extrapolation to real situations, we evaluate exposure risk under field conditions to estimate the probability of a lethal dose, showing that not all reservoirs with detectable pathogens are significant transmission pathways.

Understanding the ecological processes that drive zoonotic disease outbreaks is important in mitigating their effects on human and animal populations. One critical factor affecting infectious disease dynamics is spatio-temporal heterogeneity in transmission^1^,^2^. For directly transmitted pathogens, this heterogeneity can be described through peer to peer contact rates and the structure of social networks in host populations^3^,^4^,^5^. When pathogens are acquired from environmental reservoirs, the pathogen’s distribution in the environment and the interaction between host behavior and reservoir becomes the crucial link in transmission. Despite the global burden of diseases with environmental reservoirs (like cholera, tularemia, polio, botulism, anthrax, bubonic plague, leptospirosis, enteropathogenic *E. coli* and others^6^), microbial ecology in the field is currently understudied due to the difficulties of monitoring microbes in their natural environment. Though progress is being made in risk assessment studies, more data are needed on diseases currently posing threats to public health, livestock health, and wildlife health. Moreover, to calibrate the risk and thus an appropriate response to emerging diseases following environmental change or the release of bioweapons, we need to know their long-term behavior in natural systems.

The distribution of infectious agents in the environment is typically aggregated due to pulsed releases from infected hosts, creating heterogeneity in the risk of exposure for future hosts. Public health scientists assess risk of infection by identifying disease agents, quantifying exposure, and characterizing dose-response relationships^7^,^8^. The challenge for risk assessment in wildlife disease systems is a lack of data on natural exposure levels and dose-response relationships that link exposure to host response (i.e. infection, resistance, tolerance^9^). We often do not know where or how hosts become infected. This, in turn, leads to uncertainty as to whether the pathogen concentrations encountered are sufficient to induce an infection, and thus, which potential exposure pathways are epidemiologically meaningful at the population level.

A pathogen-centered view of epidemiology may classify host contacts as “effective” contacts (where the pathogen’s progeny are transmitted beyond the exposed host) and “dead-end” contacts (where the pathogen’s lines of descent are terminated in the exposed host). Merely detecting the presence of a pathogen in the environment does not necessarily establish a credible pathway for future infections. In particular, failure to consider dose may lead to greatly exaggerated risk estimates for pathogens such as *Bacillus anthracis* (BA), the causative agent of anthrax. BA transmission requires host death and release of bacterial spores into the environment to be contacted by a susceptible host. Thus, an effective contact is equal to a lethal contact, making this an ideal study system for exploring exposure levels through different pathogen sources and transmission pathways.

BA can be transmitted via oral, pulmonary or cutaneous routes, although in non-human animals the oral route is most common^10^. BA enters the environment from carcasses and, accordingly, carcass sites are considered likely foci for pathogen ingestion and disease transmission^11^. However, long-lived BA spores can be transported away from carcass sites by several biotic (e.g., vertebrate or invertebrate scavengers) or abiotic (e.g., water or wind) processes^12^,^13^,^14^,^15^,^16^. Watering holes have been considered potential hotspots for BA transmission^16^,^17^,^18^,^19^, because spores may accumulate in sediments from carcasses in the water, transport by vertebrate scavengers, or surface runoff, and the water serves to attract new potential hosts. However, dilution in the water column and precipitation of BA spores into sediments may reduce the potential for waterholes as BA transmission hotspots when infectious dose is taken into account.

Several lacunae, typical of many disease systems, exist in our understanding of anthrax epidemiology. One is that experimentally determined lethal doses (LD; Table S1) appear to be several orders of magnitude higher than BA concentrations measured in the environment^20^ (Table S2). Oral infection experiments for BA have been conducted mostly as part of vaccine trials, where the goal was to find a single dose that would kill 100% of control animals as a test of vaccine efficacy (Table S1). From these studies we can estimate that an oral challenge of 10^8^ spores would approach 100% lethality. Attempts to reconcile differences between LDs and environmental concentrations have emphasized the inherent differences between natural environments and laboratory settings and the limitations of extrapolating results from the laboratory to nature^10^. Indeed, host susceptibility is suspected to be an important source of transmission heterogeneity, and can vary based on behavior, age, sex, reproductive status, prior exposures, general immunological status, and genetics^21^,^22^,^23^. Accordingly, we have observed variation among hosts in exposure and susceptibility to BA^11^,^24^,^25^,^26^,^27^. Estimates of a minimum LD would be relevant for tracking BA transmission in natural populations, but are not known and will vary among species, among individuals and over time.

Here we attempt to bridge this gap through an integrated approach that combines new estimates of BA concentrations in the environment with insight into animal behavior, while including the effects of LD heterogeneity. We model exposure dose based on herbivore rates of ingestion, evaluating how both pathogen concentration in the environment and host exposure risk vary over time. These models are based upon behavioral data for important anthrax hosts in Etosha National Park, Namibia. These include plains zebra (*Equus quagga*), the most common anthrax host, and African elephant (*Loxodonta africana*), the largest bodied host capable of the greatest rates of daily ingestion. These models enable us to integrate the component pathogen datasets with host behavior to assess fundamental parameters of disease ecology from a new, holistic perspective. We quantify concentrations of BA at carcass sites and watering points (Fig. 1), the two most probable sources of host-pathogen contact. At carcass sites we examine where BA is concentrated on grasses and in soils, and factors affecting BA long-term survival. Using sediments from water sources, we assess BA concentrations in seasonal and perennial sources. Our aim is to highlight complexities of ecological interactions that often need to be discovered in natural settings before they can be teased apart in experimental settings. We also demonstrate how field observations can be used to “ground truth” the results of laboratory studies that test components of natural disease systems.

## Results

### Host exposure to BA through grazing at carcass sites

We found exponential decay in BA concentrations at carcass sites in soils and on grasses (Fig. 2), though with interesting and yet unresolved variance among years and sites, and with BA at easily detectable concentrations in soil four years after death. When controlling for differences in sample mass and site age, no significant differences in colony forming units (CFU)/g could be found among grass species or any soil characteristics (all *p* > 0.1; soil characteristics summarized in Table S3). Detecting no evidence of soil properties on BA persistence among sites may reflect the similarity in soils across the study area. We found a strong positive relationship between the estimates of BA concentrations in soils from culture and qPCR (*t* = 7.81, *p* < 0.0001, *R*^2^ = 0.504, *N* = 62 sites), although the estimates diverged from a 1:1 line as concentrations increased beyond approximately 10^4^ CFU/g (Fig. 3) and our DNA extraction efficiency from spores was low at 7.8 ± 5.7% (additional information in SI Results).

BA concentrations found on grass above-ground components were significantly lower than on the roots or in the soil (top versus soil: *p* < 0.0001, base versus soil: *p* = 0.0483, root versus soil: *p* = 0.7337; Fig. 2), in agreement with Turner *et al*.^11^ Separating the above-ground component into tops and bases indicates that BA spores are concentrated more at grass bases, near the soil surface, than further up the plant (tops versus bases: *p* = 0.0004, Fig. 2). However, by far the highest BA concentrations are found in the surface soils and the soils associated with grass roots (Fig. 2).

The grazing simulation models suggest that there is a significant risk of infection from feeding at carcass sites 0–2 years after death, even if little grass is consumed (Fig. 4). It is notable that this high risk of infection emerges even without accounting for direct ingestion of roots or soil. If small amounts of soil are ingested—through behaviors widely documented in herbivores^27^—the risk becomes even greater and longer lasting (Fig. 5). Including a modest component of soil or root ingestion in the grazing simulations prolongs the likely transmission period into the fourth year after death (Fig. 5), and possibly longer. We also explored what effect ingesting different parts of the above-ground biomass component (roughly reflecting foraging height) might have on model outcomes and host exposure (see SI Results), however, additional research would be required to investigate this more thoroughly.

### Host exposure to BA through drinking

**Figure 1:**
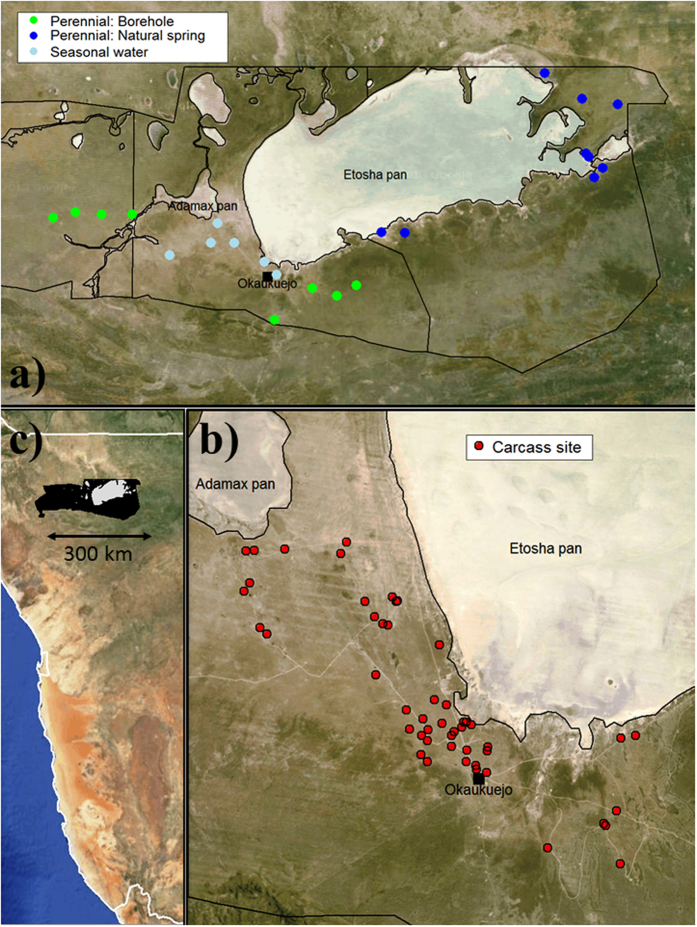
Study areas. (**a)** Central and eastern Etosha National Park showing locations of all sampled water sources (light blue: seasonal waterholes, green: perennial boreholes and blue: perennial natural springs). (**b)** Monitored carcass sites (red) in central Etosha National Park, in relation to features such as salt pans. (**c)** The location of Etosha National Park (black) in northern Namibia. Maps were made using the packages “shapefiles”^59^ and “maptools”^60^ in R version 3.1.1 (www.r-project.org), with satellite images from NASA’s Visible Earth Project (http://visibleearth.nasa.gov).

The only robustly significant predictor of sediment CFU/g was the type of water source, with seasonal sources containing significantly higher BA concentrations than natural springs or boreholes (overdispersed Poisson GLM multiple regression p < 0.01; Table S4). Given the unavoidable spatial aggregation of water sources sampled (Fig. 1), we cannot determine whether these differences reflect physical properties of these water types or larger-scale landscape patterns in anthrax incidence. The other candidate variables we evaluated were sediment condition at the time of sampling (wet versus dry) and seasonal/long term trends. Although detecting seasonal trends in BA concentrations was a goal of the waterhole study, no significant patterns were detected, and sediment condition showed no significant influence.

The exposure model suggests that risks of an LD through drinking may only occur when very high water intake is combined with very high host susceptibility (i.e. low LD), and high transfer rates (i.e. high α, see Equation 4; Fig. 6; Figure S1). For example, if *α* = 10^–3^ the probability of infection is assessed as being greater than 0.001 only if LD < 10^5.7^, to exceed 0.01 only for seasonal water sources when water intake >25 L, and never for natural springs or boreholes. An *α* = 1 value is highly unlikely to occur in nature, where the concentration of BA in the water column (CFU/ml) equals the concentration in sediments (CFU/g). Even under this worst-case scenario, a low (1L) water intake implies a probability <0.001 of infection, or even 0.1 if LD < 10^6^. On the other hand, if LD > 10^6^, larger water intakes would have to occur for an LD to happen with epidemiologically significant frequency. Thus, the question of whether water sources are important for anthrax depends not on whether the presence of BA can be demonstrated in waterhole sediments, but on the LD, the ingested quantities of water, the behavior of hosts (i.e. the amount of sediment disturbed by drinking animals), and the physical properties of BA in the water column.

## Discussion

This study highlights challenges in understanding disease transmission when information about exposure risk and dose-response relationships is limited and the importance of different transmission pathways is unknown. We demonstrate that such an integrated approach may be the best way to address critical knowledge gaps in the ecology of disease transmission when experimentation, for ethical and practical purposes, cannot be conducted. In particular, studies for virulent pathogens run the risk of extrapolation when experimental studies are untenable (e.g., determining LDs for large mammals), or fail to capture critical epidemiological factors (e.g., interactions with soil microbiota or plants not present in the lab), and we show that studies under field conditions offer alternative risk assessment approaches.

Animal dose-response models for the inhalational form of anthrax show a sigmoidal response with an LD_50_ (a dose that kills 50% of a test population) falling between 10^4^–10^5^ spores^28^,^29^. Existing data for gastrointestinal anthrax (Table S1) are insufficient for developing dose-response models, although we can expect that an LD_50_ for this comparatively less deadly form of anthrax would fall within the range of 10^5^–10^7^ spores. However, the ultimate goal is not to estimate an LD_50_, but to determine epidemiologically plausible values for exposure and an infective threshold that would maintain the disease in a system, and enable prediction of transmission dynamics based upon observed mortality rates.

With this study we have shown that carcass sites are more likely to be important sources of host-pathogen contacts than water sources. At carcass sites, a spike in the probability of contacting an LD occurs at exposures between 10^5^–10^6^ spores, and persists for the length of the dataset. This spike is observed whether hosts ingest purely above-ground biomass (Fig. 4) or also a portion of roots and soil (Fig. 5). At carcass sites of ages 0–2 years, individuals grazing there are very likely to ingest large doses of BA spores, even if very little grass is consumed. The probability of lethal exposures declines with time, yet is still not insignificant even at lower probabilities, given that these sites are frequently visited and grazed by herbivores^11^. This may contribute to endemic cycles or to annual spillover events, where smaller outbreaks occur in the years following a large outbreak. The exposure model suggests that any grazing at carcass sites in year 0 is very likely to result in death, unless the LD is very high and very little grass is consumed (Fig. 4). Thus, the main factor decreasing the importance of year 0 transmissions may be the denuding of grass biomass associated with scavenging of the carcass^11^, since anthrax mortalities in Etosha peak toward the end of the rains and sites may remain bare of vegetation until the following rainy season. Transmission seems likely to be most frequent in years 1–2 when grass regeneration has occurred and BA concentrations remain substantial. Transmission in years 3–4 is comparatively less likely, suggesting that the main effect of an anthrax outbreak on host populations will be with a lag of 1–2 years, producing a delayed density response on their population dynamics.

Variation in pathogen persistence in the environment can strongly influence outbreak dynamics and frequency^30^,^31^,^32^. Thus, the rate of decay in spore concentrations may influence the between-outbreak interval among systems. In areas with sporadic outbreaks, a decade or more can pass between subsequent outbreaks^33^. It is difficult to predict how carcass sites may drive outbreaks in systems with such large inter-outbreak dynamics. In general, anthrax outbreaks have been linked to rainfall extremes^34^. One may hypothesize that shortly after death rainfall increases transmission events by encouraging grass regrowth and foraging at carcass sites, but that as years pass and little BA is found on grasses, drought might increase the risk of restarting an epidemic from old carcass sites if depleted biomass leads herbivores to ingest more soil when foraging. Thus, old carcass sites may only sporadically contribute cases that are negligible during active infection cycles but that could restart an outbreak years after the disease had seemingly disappeared locally.

**Figure 2:**
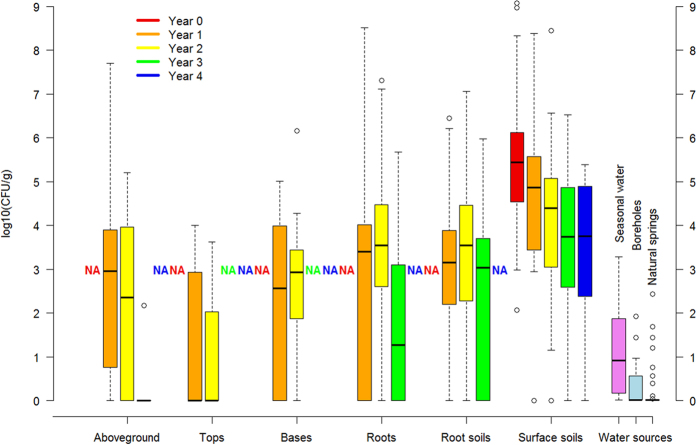
Concentrations of *Bacillus anthracis* in log_10_ colony forming units per gram (CFU/g) at carcass sites (0–4 years after host death) and at water sources. The black line is the median value, the upper and lower boxes represent the quartile range of 25–75% of the data, and the whiskers represent the 95% confidence interval; outliers are shown as open circles. “Bases” are the lower 1cm of the above-ground component of the grass, “tops” are the remainder of the above-ground component, (thus “tops” + “bases” = “aboveground” biomass component), “roots” are the below-ground component and “root soils” are the soil surrounding each plant. “Surface soils” are the top 1cm of soil collected 0–1 m from the center of the carcass sites. “Water sources” are sediment samples collected below seasonal waters (gravel pits or natural pans) or perennial waters (boreholes and natural springs).

**Figure 3:**
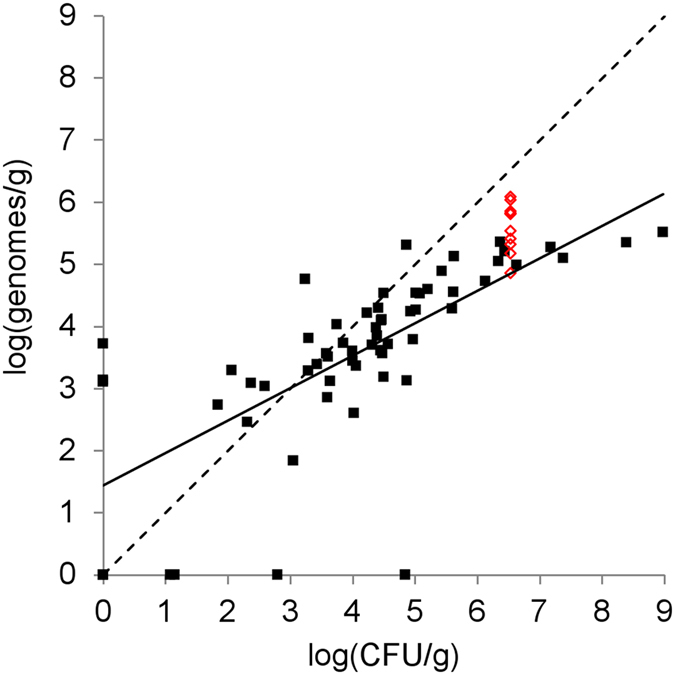
The correlation in *Bacillus anthracis* concentrations from carcass soil samples estimated by bacterial culture (in CFU/g) and by qPCR (in genomes/g). Both estimates are corrected for soil moisture. The dashed line is the 1:1 line and the solid line a fit to the data. *N* = 62 sites, black squares. The results from the soil spiking experiment are shown in open red diamonds.

**Figure 4:**
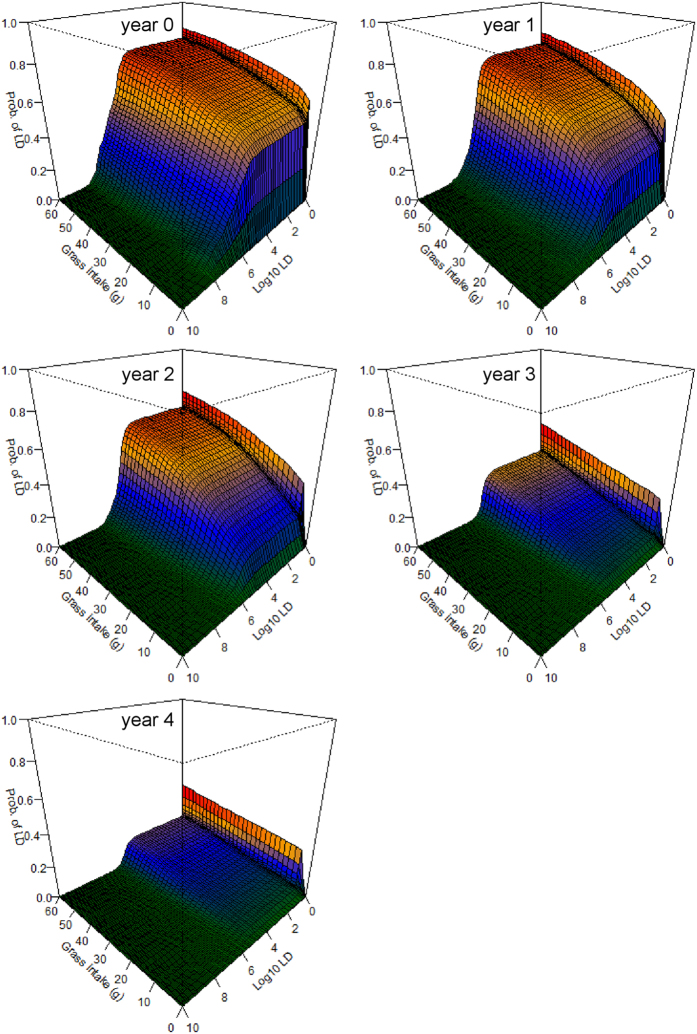
The probability of ingesting a lethal dose (LD) of *Bacillus anthracis* (BA) from grazing at anthrax carcass sites 0–4 years after host death. The horizontal axes denote the amount of grass biomass ingested from a site (from 0–60 g) and a range of potential LDs of BA (from 1–10^10^ spores on a log_10_ scale) respectively, while the vertical axes show the resulting probability (from 0–1) of exceeding that LD based on the amount of grass biomass ingested. The colors are a heat map to denote relative risk within (not among) figure panels. These models assume only above-ground grass biomass is ingested, with no additional soil or root contact (Equation 2).

**Figure 5:**
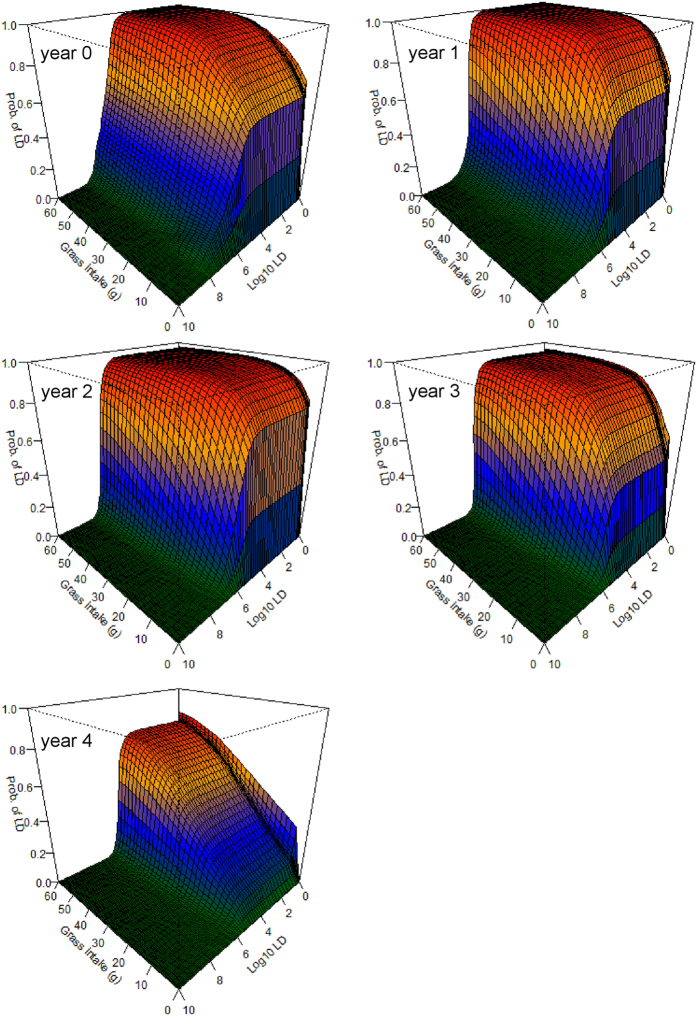
The probability of contacting a lethal dose (LD) of *Bacillus anthracis* (BA) from grazing, and ingesting soil, at anthrax carcass sites 0–4 years after host death. The horizontal axes denote the amount of grass biomass ingested from a site (from 0–60 g) and a range of potential LDs (from 1–10^10^ spores) respectively, while the vertical axes show the resulting probability (from 0–1) of exceeding that LD based on the amount of grass biomass ingested. The colors are a heat map to denote relative risk within (not among) figure panels. These models assume 90% of the mass ingested is above-ground grass biomass, with 10% consisting of grass roots with an equal mass of soil attached (Equation 3). Including a component of root and soil ingestion in the models results in infection risks that are markedly higher for a considerably longer time than those seen in Fig. 4.

**Figure 6:**
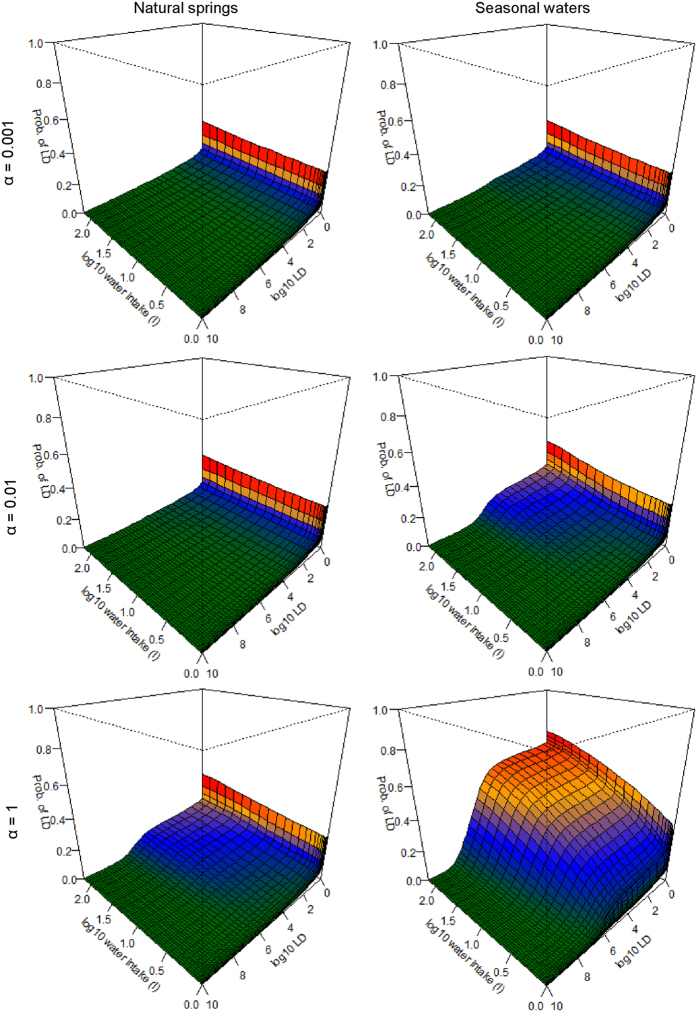
The probability of ingesting a lethal dose (LD) of *Bacillus anthracis* (BA) at natural springs and seasonal waters. The axes and colors are consistent with Figs 4 and 5. The intake axes denote water ingestion from 1–250 L. BA concentrations were measured in sediments, and how much BA may be found in water is unknown, but can be assumed to be much lower than sediments. Therefore we show a range of possible exposures, where the amount in water is 1/1000^th^ what is found in sediments (*α* = 0.001), where the amount in the water is 1/100^th^ what is found in sediments (*α* = 0.01), and an unrealistic, worst-case scenario where the amount of BA in the water is the same (per unit mass) as the amount in the sediments below (*α* = 1) (Equation 4). Results for boreholes are similar to natural springs, and are shown in Figure S1.

Water sources show a much lower potential to transmit lethal infections than carcass sites. Seasonal water sources held significantly higher concentrations of BA in sediments than did perennial sources (Fig. 2); however, these concentrations were still orders of magnitude below those observed at carcass sites. Water sources are likely to contribute lethal exposures only with a combination of i) an extremely high water intake, ii) a very high transfer rate (which probably varies with animal behavior) and iii) if the threshold for a lethal infection is considerably lower than previously thought (Fig. 6). Etosha is an example of a system that should have the greatest potential for drinking-based transmission of BA. Point water sources aggregate hosts and have the potential to aggregate the pathogen as well, whereas streams or rivers have a lower potential to aggregate host and pathogen for transmission. Thus, ingestion of contaminated water may not be a significant transmission pathway for BA across systems. An exception to this may occur for semi-aquatic species such as hippos that exhibit considerably different risk behaviors for water-borne transmission^35^.

One surprising result of this study is that concentrations of BA on roots and root-associated soils seemed to increase two years after death, a pattern seen across sampling years, while concentrations in other sample types exhibited the expected decay over time (Fig. 2). Why such a response would be observed two years after death, and not in the first growing season, is unknown. Despite great interest in Van Ness’s incubator area hypothesis proposing BA replication occurs in the environment^36^, and what role an off-host growth phase would play in epidemiology, BA replication in the environment has so far not been detected. Recent experiments do demonstrate interactions between BA and grasses^37^,^38^, evidence that the pathogen may have a far more complex lifecycle in the environment that previously understood. However, future research is required to determine mechanisms behind this observed increase in BA (e.g., soil-water dynamics aggregating spores around plant roots or BA population growth), and what role BA may have in the grass rhizosphere and soil microbial community.

For environmentally transmitted pathogens, there are several challenges that currently prevent a full understanding of the sources of transmission heterogeneity and their role in disease dynamics. From the pathogen perspective, the outcomes of host-pathogen contacts may be binary (infections continue or not), but the threshold is a moving target. In this paper and in the existing dose-response literature for gastrointestinal anthrax, dose is considered as a one-time event. However, immune signatures of sublethal exposures are observed in nature^24^,^39^, and we can therefore expect that animals in endemic areas will encounter low dose, non-lethal exposures. The complexities of individual dose size during exposure to a single infectious site versus the total dose acquired over time in sequential exposures remains unknown on disease outcomes for gastrointestinal anthrax. In *Cryptosporidium parvum*, for example, as length of exposure time increases, the probability of infection decreases, even with variable dose magnitudes^40^. Thus sequential low-dose pathogen exposures could increase the threshold for a lethal exposure in endemic versus sporadic disease systems. These complexities highlight how disease management may be hindered, if based on simple pathogen presence/absence studies or unrealistic dose-response relationships of little relevance to natural systems.

Our analysis encompasses a systems-level approach to meet challenges in studying transmission of an environmental and zoonotic disease. Crucial epidemiological and risk-relevant data are hard to come by for highly controlled pathogens like BA, because there are obvious limitations to studying environmentally transmitted diseases solely in laboratory settings. Hence, the study of unmanaged BA in its natural habitat offers otherwise inaccessible insights into the world of virulent zoonotic diseases. Our study quantifies exposure risk in a heterogeneous environment, which is a crucial step in risk assessment, but our study also has further implications. For example, coupling surveillance-based data on mortality, immunology, and host movement with the exposure estimates presented here will allow us to approach the dose-response equations without animal experiments. Fortunately, with the emergence of cheaper and quicker molecular tools, increasing density of remotely sensed data and computing power, the data-intensive and cross-disciplinary work needed to achieve what amounts to microbial population dynamics in real systems is increasingly feasible. Thus, the approach used in our study takes a pathogen-centric view of the epidemiology of environmental diseases, leading to a deeper ecological understanding of outbreak dynamics, which should lead to better risk assessment and disease management. This is important for using intervention resources effectively to target the most important transmission pathways; in our example, grazing at carcass sites 0–2 years after death as opposed to drinking at waterholes.

## Material and Methods

### Study area

This study was conducted in Etosha National Park, a large, semi-arid savanna system in northern Namibia. Etosha has a primarily flat terrain, with a large, barren, salt pan covering approximately 1/5 of the area of the Park. The pan is surrounded by grasslands and dwarf shrub savannas that attract large herds of herbivores. Soils across Etosha are consistent with properties of semi-arid soils, including weak profile development, low organic matter, and low to moderate fertility^41^. The soils on the central Etosha plains (where most anthrax cases are observed) are high in calcium carbonate, and of moderate to high alkalinity, salinity and drainage^41^. Rainfall is strongly seasonal and three seasons are recognized in Etosha: cool dry (May–August), hot dry (September–December), and hot wet (January–April, when most anthrax cases are recorded)^27^. The only perennial water in the park comes from point water sources. These include boreholes, where groundwater is pumped into a depression creating a man-made waterhole (sometimes via a trough that overflows into the waterhole, sometimes directly into the waterhole), or from natural artesian or contact springs situated primarily along the pan’s southern edge^42^.

Although anthrax has been recorded throughout the park, the grassland plains of central Etosha to the west of the pan are where the most cases are observed. This area has few perennial water sources; thus large herds of herbivores are distributed throughout these plains when seasonal water is available, and there is a migration eastward along the southern boundary of the pan as conditions dry out^43^. Animals do remain on these central plains year round, but their distributions contract to the areas within reach of perennial water. The dominant anthrax host in Etosha is plains zebra, accounting for >50% of recorded mortalities, followed by blue wildebeest (*Connochaetes taurinus*), springbok (*Antidorcas marsupialis*) and African elephant^27^.

Anthrax is considered part of the ecosystem and is not actively managed^44^, a perspective that allows for the study of transmission dynamics in the natural environment. Samples for BA quantification were collected at zebra anthrax carcass sites and at seasonal and perennial water sources over a five-year period from 2009–2014 (Fig. 1). Although standardized methods for identifying and quantifying the pathogen are desirable^45^, different methods were used for samples from carcass sites and water sediment samples, due to challenges of working with materials with very different pathogen concentrations. At carcass sites the concentrations of BA were high enough allowing reliable quantification using the “gold standard” for BA, culture in serial dilution^10^. We then conducted qPCR on a subset of carcass soil samples for BA DNA extracted directly from soil, as a validation of our bacterial colony identifications on a culture media that is only semi-selective, and as a test of whether quantitative results from culture and qPCR correlate, despite the anticipated low DNA extraction efficiency from BA spores^46^,^47^. For sediments from water sources, preliminary analyses indicated that BA concentrations were low and quantification via culture unreliable, hence we developed and validated an alternative quantification procedure, involving a growth step in culture followed by qPCR on DNA extracted from vegetative cells. The specific methods employed at carcass sites and water sources are detailed below.

### Sample collection at carcass sites

Sampling was conducted in central Etosha from 2010–2014 at 41 adult (≥2 years) zebra carcass sites. These sites include *N* = 26 from 2010, *N* = 4 from 2011, *N* = 8 from 2012 and *N* = 3 from 2013 (details of how sites were selected and marked are in SI Methods). All sites were anthrax positive carcasses, with the exception of one 2010 carcass site that served as a negative control. Blood swabs of carcasses were tested for BA through culture with PCR confirmation^48^.

Soil samples from the year of death (year 0) were collected 0–2 months after death. Thereafter, sites were sampled annually, from February–April, during peak anthrax mortality^11^,^27^. Herbivores are most likely to contact the soil surface; therefore we sampled the top 1cm of soil. A sterile spoon was used to scoop sub-samples of soil within 1m of the site center, for a total of ~25 g of soil. Additional soil samples were collected to test the effect of soil properties on BA concentration over time. However, as none of the recorded soil characteristics affected persistence, these data are described and summarized in SI Methods.

Grass sampling follows the methods of Turner *et al*.^11^, and data collected in 2012–2013 from that study are combined with new data collected in 2014 to build the grazing exposure models. New sampling deviated from the prior by testing for species differences among grasses sampling 2–3 species/site rather than just the single dominant species (with 3 replicates/species/site collected in all sampling years), and by dividing the above-ground grass component into two samples (tops and bases) for culture. Grazers rarely consume the entire above-ground portion when feeding and we hypothesized that BA may occur in higher concentrations proximate to the soil surface. Thus each plant sampled in 2014 was divided into base (the lower 1cm of the above-ground component), top (the remainder of the above-ground component), roots, and surrounding soil, and each of these four sample types was cultured separately. Since the prior study showed that BA spore concentrations on grasses dropped to near zero three years after death, grass sampling in 2014 was conducted only at carcass sites 1–2 years old (*N* = 8 sites). In total, we had grass data from *N* = 10 1-year old sites, *N* = 16 2-year old sites, and *N* = 6 3-year old sites, representing data from 140 individual plants and their soil.

The concentration of BA in soils and grasses was assessed via bacterial culture adapted from standard soil protocols^10^,^11^,^38^. Five grams of homogenized soil was combined with 45 ml 0.1% sodium pyrophosphate in a sterile 50 ml centrifuge tube. These mixtures were vortexed for 10 minutes to dislodge spores from soil and then centrifuged at slow speed (300 *g*) for 2 minutes to settle soil particles. The supernatant was centrifuged at 3000 *g* for 15 minutes to pellet spores and residual soil. The pellet was resuspended in 5 ml of aqueous carrier and 1ml was used to create serial dilutions from 10^−1^ to 10^−6^. 100 μl of the dilution series was plated directly onto PLET (polymyxin-lysozyme-EDTA-thallous acetate) agar, and incubated at 30 °C. Grass samples were cultured following the soil protocol, with the entire sample combined with sodium pyrophosphate.

Colonies of BA were identified morphologically, with colony counts conducted after two and four days. In cases where identity was uncertain, the colony was sub-cultured for BA confirmation tests^10^. Counts of CFUs were adjusted to represent CFU/g of dry matter. Samples were given coded identification numbers for culture, and the negative control site was culture negative at each sampling point.

To compare and validate quantitative data obtained from culture methods, DNA was extracted from a subset of the carcass site surface soil samples for qPCR (DNA was extracted from 62 soil samples, with 48 of these extracted in replicate to compare repeatability of our qPCR results). Approximately 0.5 g of soil was extracted, with the precise weight recorded to standardize BA concentration/g of dry soil. DNA was extracted using FastDNA™ Soil kits (MP Biomedical). The manufacturer’s protocols were followed except that samples were bead-milled for 3 minutes at maximum in a Mini-Beadbeater-16 (Biospec Products). To evaluate the DNA extraction efficiency for this protocol, BA-free soils (*N* = 5 samples) from across the study area were spiked (in duplicate) with Sterne strain 34F2. 0.5 g soil was placed directly into the extraction kit tubes and spiked with 3.4 × 10^6^ spores (100 μl undiluted preparation, Onderstepoort Biological Products) and then followed the DNA extraction protocol. For quantification of BA in the extracted soils, the plcR gene (a single copy gene) was targeting using taqMAMA, run according to Easterday *et al*.^49^ using a LightCycler^®^ 96 (Roche) (details in SI Methods).

### Sample collection of sediments at water sources

Sediments from 23 water sources located throughout Etosha were surveyed for BA (Fig. 1). These comprised six seasonal water sources (gravel pits or natural low-lying areas), and 17 perennial water sources (natural springs and man-made boreholes). Sites were sampled sporadically over time, collecting samples across the different seasons of Etosha. At the most regular, sites were sampled every two months from November 2009–March 2011 with a further wet season sample in 2012 and 2013, and at a minimum some sites were sampled only 1–2 times due to challenging accessibility (see Table S4 for further detail on sample numbers/site). None of the sampled gravel pits were actively used during or immediately preceding the sample period, so while they are man-made sources, their disturbance patterns are similar to natural seasonal sources.

Specific sampling locations were selected at the deepest accessible points where water, and potentially spores, could concentrate during drying periods, and were revisited based on GPS coordinates. At each location 50–100 g of sediment was collected. Sediment samples were collected in cores of at least 10 cm depth to sample the sediment most likely to be churned up by drinking animals. When a sampling location was dry this was done with a soil core sampler; when a location was under water, sediment samples were collected using a shovel-like device (Figure S2). Deep locations were selected as the locations in water sources likely to contain the highest concentrations of BA, based on the hydrodynamics proposed in the water concentration hypothesis of Dragon and Rennie^50^.

Initially we attempted to quantify BA in these samples using standard protocols. However, low concentrations of the pathogen coupled with high background fauna of other spore-forming bacteria made detection and quantification in culture unreliable. Thus we used qPCR to detect and quantify BA in sediments. Due to the low DNA extraction efficiencies expected for BA directly from spores in soil^46^,^47^ (from factors such as attachment of DNA to soil particles, incomplete spore lysis, and varying inhibition), we first performed a growth step on culture media to promote germination of spores. This improved our ability to extract DNA (from vegetative cells) and thus detect low quantities of BA using PCR. This combined culture-PCR method was developed and employed by Beyer *et al*.^48^ but was only used for detection, not quantification (Letant *et al*.^51^ also employed a culture-PCR method for detection of low BA quantities from soil using different protocols). We present experiments validating our method for quantifying low concentrations of BA in sediment samples (see SI Methods) and employ it to quantify BA in waterhole sediments.

Two grams of sediment was suspended in 4–6 ml of water, vortexed thoroughly and 1ml of the suspension spread onto four TSPBA (trimethoprim-sulfmethoxazol-polymyxin-blood-agar) plates. After overnight incubation at 37 °C, bacterial lawns were scraped off using 1ml water per plate. The suspension was heat treated at 110 °C for 20 min, centrifuged at 4000 *g* for 20 min, and 5 μl of the supernatant used for PCR targeted to the PA gene. qPCR was performed using the protocol detailed in W.H.O.^10^ with the PA-S and PA-R primers from Ellerbrok *et al*.^52^ PCR Ct values were linked to spore counts by spiking known quantities of spores to soil samples (see SI Methods).

### Data analysis and exposure models

The carcass site BA concentration data (CFU/g dry matter) were split into samples from aboveground (grass inflorescences, leaves, stems) versus belowground (root, surrounding soil, and soil surface samples) (see SI Methods). Where grass data were lacking, generalized additive models (GAM)^53^ were used to model above-ground grass BA CFU/g from the surface soil data, in order to estimate BA concentrations on grasses from sites where grasses had not been sampled. GAMs were used when possible to avoid constraining results by assumptions about parametric functions, but the degrees of freedom of each smoothing term were limited upwards to four to avoid overfitting and only allow unimodal effects. There were strong correlations between the CFU/g dry matter found in soil, root and above-ground grass samples from the same site and time. A simple regression model on the form


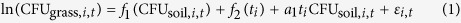


where *ε* is the error at site *i*, at time *t* (in years since death of host) and independently drawn from a quasi-Poisson error distribution allowing for overdispersion, explains about 76% of the deviance in the response variable. This correlation was deemed sufficient to estimate BA concentrations on grasses based on the concentrations of BA in the surface soil. The predicted values were then substituted for missing grass values to get an unbiased estimate of combined above-ground grass CFU/g. Thus, whenever grass samples did not exist but surface soil samples did (mostly from years 0 and 4) they were substituted with above-ground grass CFU/g estimates from surface soil concentrations and site age. Exposure simulations used this combined set of raw and predicted data.

We built the grazing exposure model based on plains zebra foraging behavior, because this species is the most common anthrax host in the system and has the strongest attraction to foraging at carcass sites^11^. To estimate zebra grass intake, and hence BA exposure, when feeding at a carcass site, we used 60 g of grass as a biologically realistic maximum intake per site. This is based on a study showing zebra grass intake to be on average approximately 6 kg/day^54^ and measurements of available grass biomass at carcass sites and host foraging behavior at camera-surveyed sites^11^. Given the relatively low productivity of a semi-arid system and the long distances zebra roam per day, it is unlikely that animals would consume more than 10% of their daily intake (i.e., our 60 g threshold) from a single carcass site. The exposure simulation model assumed each bite of grass is 2 g biomass on average^55^, and that an animal can graze from 0–60 g of grass from a site— and an individual only encounters a single infectious site (we do not address multiple “stacking” exposures in this study).

To simulate pathogen exposure while grazing at a particular site, doses were bootstrapped. This was done by resampling 1000 carcass sites of each age class and then drawing *n* samples from each site age, with *n* being the number of 2 g “bites” needed to ingest a certain amount of biomass (i.e. 1–30 bites for 2–60 g grass). By treating each “bite” as a Poisson process (denoted “Pois[argument]” below), the exposure *D* from ingesting *g* grams of aboveground grass at a carcass site *i* of age *t* was assessed as


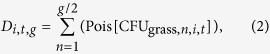


where *n* is the individual sample of CFU/g BA drawn from the observations of CFU/g in aboveground grass biomass at site *i* at age *t*. Likewise, when a component *β* denoting weight proportion of roots with equally increased soil ingestion was included, this was given as


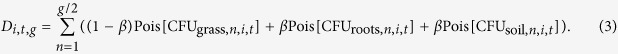


The integrals of the empirical density distributions of the simulated exposures were then used for calculating the probability of a grazing pass resulting in an exposure exceeding the LD. Because LD is unknown, we explored a range of values from 1–10^10^ spores, which encompasses minimum and maximum exposures likely to be encountered in nature and the reported experimental LD of 10^8^ spores (Table S1). Thus we get infection probabilities as a function of grass biomass ingested, LD range and age of site (years since host death). Although *β* is unknown, we know *β* > 0 since zebras will ingest roots/soil when foraging (W.C.T. personal observation). However, since they primarily consume above ground biomass, it seems likely that *β* < 0.5. To get a conservative estimate of the effect of soil ingestion, we report the results of an arbitrarily chosen value of *β* = 0.1 in our models.

In addition to BA exposure from the above-ground component of grasses, herbivores are known to ingest soil^27^ and may thus be exposed to additional BA from soils or plant roots. Because some of this soil is already reflected in soil particles on the above-ground component, and geophagia may occur separately from grazing^56^, we first ran the model where exposure occurred only through consumption of above-ground biomass (Equation 2). This represents a conservative model of grazing exposure, because soil does adhere to roots, and plants can be plucked and eaten whole while grazing, including roots and attached soil particles. Since the prevalence and variation in this behavior is unknown, the model was rerun to visualize the effect of including small amounts of roots with adhering soil (Equation 3). Here a percentage *β* of the forage weight is roots, with an equal weight of surface soil particles. The effect of this term is illustrated using 10% as a value for *β*. All analyses were implemented using the R statistical software^57^.

We built the drinking exposure models based on drinking behaviors for zebras and elephants, with elephants representing the maximum amount of water that could be ingested by any potential host species. BA CFU/g of sediment from water sources were estimated from the PCR standard curve of Ct-values (see SI Methods). The drinking exposure risk model is a function of the unknowns LD and intake at a water source, spanning biologically plausible ranges as for the grazing models and assuming daily intake is from a single water source. Individual zebras drink an average of 14 L/day and elephant males 89 L/day (Young 1970 cited in Gaylard *et al*.^58^). Considering individual variation and time intervals between drinks, we estimated that zebras could drink up to 24 L/day and male elephants up to 240 L/day. Thus we explored the range of 1–250 L/day. The estimated CFU/g in sediment samples were then analyzed using multiple regression models in a generalized additive framework, allowing for overdispersion in the residuals (see above). The exposure model for water sources assumes a relationship between the BA concentrations in the sampled sediment (CFU_g sediment_) and the slurry of water and sediment actually encountered by drinking animals (CFU_g water_) where CFU_g water_ = *α* CFU_g sediment_ where the transfer rate *α* is likely 0 < *α* < 1. Thus, if an animal ingests *L* liters of water from a waterhole in a day, total exposure,


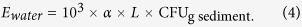


To simulate exposure we thus resampled sediment samples and made each the Poisson mean centered on its CFU value for each sample, divided the resulting values into classes depending on water type (seasonal/borehole/natural spring) and integrated the area of the resulting empirical density distributions above the LD over the range as for the grazing models.

## Additional Information

**How to cite this article**: Turner, W. C. *et al*. Lethal exposure: An integrated approach to pathogen transmission via environmental reservoirs. *Sci. Rep*. **6**, 27311; doi: 10.1038/srep27311 (2016).

## Supplementary Material

Supplementary Information
