# Supplementary material for: Lethal exposure: An integrated approach to pathogen transmission via environmental reservoirs
Source: Sci Rep. 2016 Jun 6;6:27311. doi: 10.1038/srep27311 (PMC4893621; doi:10.1038/srep27311)
Supplement: Supplementary Information [file srep27311-s1.pdf]

Supplementary Information

Lethal exposure: An integrated approach to pathogen transmission via environmental reservoirs

Wendy C. Turner[1,2,3*†], Kyrre L. Kausrud[1†], Wolfgang Beyer[4], W. Ryan Easterday[1], Zoë R. Barandongo[5], Elisabeth Blaschke[4], Claudine C. Cloete[1,5,6], Judith Lazak[4,7], Matthew N. Van Ert[8], Holly H. Ganz[3,9], Peter C. B. Turnbull[10], Nils Chr. Stenseth[1], and Wayne M. Getz[3,11]

[1]Centre for Ecological and Evolutionary Synthesis (CEES), Department of Biosciences, University of Oslo, P.O. Box 1066 Blindern, 0361 Oslo, Norway
[2]Department of Biological Sciences, State University of New York, Albany, New York 12222, USA
[3]Department of Environmental Science, Policy and Management, University of California, Berkeley, 137 Mulford Hall, Berkeley, CA 94720-3112, USA
[4]Institute of Animal Sciences, Department of Environmental and Animal Hygiene, University of Hohenheim, Hohenheim, Germany
[5]Department of Biological Sciences, Faculty of Science, University of Namibia, Windhoek, Namibia
[6]Etosha Ecological Institute, Ministry of Environment and Tourism, Etosha National Park, PO Box 6, Okaukuejo, Namibia
[7]Institute of International Animal Health, Free University of Berlin, Königsweg 67, 14163 Berlin, Germany
[8]Emerging Pathogens Institute, University of Florida, Gainesville, FL, USA
[9]Genome Center and Department of Evolution and Ecology, University of California, Davis, CA, USA
[10]Salisbury, UK
[11]School of Mathematical Sciences, University of KwaZulu-Natal, Private Bag X54001, Durban 4000, South Africa

[†]these authors contributed equally

Contents:

Supplementary Methods:
Carcass site selection
Soil characteristics and effects on BA persistence
qPCR protocol for carcass surface soil samples
Estimating a standard curve for water sediment Ct-values
BA on grasses data processing

Supplementary Results:
Quantifying BA in carcass site soils comparing culture to qPCR
Effect of grazing different portions of the above-ground biomass on exposure

Supplementary Tables and Figures:
Table S1: Experimentally assessed oral lethal doses of *Bacillus anthracis* spores
Table S2: Concentrations of *Bacillus anthracis* found naturally in the environment
Table S3: Average soil characteristics in the study area
Table S4: Water sources sampled for BA in Etosha National Park, Namibia
Figure S1: The probability of ingesting a lethal dose (LD) of *Bacillus anthracis* (BA) from drinking at boreholes
Figure S2: Sampling protocol for sediments below water sources

References
Those cited in online supplementary information

Supplementary Methods

Carcass site selection

All anthrax positive adult zebra mortalities recorded in the area were considered for this study; potential sites were excluded if 1) the carcass was closer than 15m to a road, 2) the carcass was in the piosphere (zone of denuded/trampled vegetation) around a watering point, or 3) the carcass was dragged by scavengers so that body fluids were released in multiple locations. Of the potential carcass sites visited, 74.1% were marked; the others were rejected because of multiple scavenger feeding locations (13.0%), location within the disturbed area near watering points (7.4%) or proximity to roads (5.6%). Sites were examined for potential inclusion in the study at least a week after death, to allow time for scavengers to consume the carcass and remove many of the larger bones from the site. Sites were located between 30m and 1.5km from the nearest road. Although anthrax carcasses may hemorrhage blood from body orifices, scavengers open carcasses first from the soft tissue around the anus and groin, depositing much of the digesta and body fluids into the soil from these exit points. This area of fluid concentration was designated as the center of the carcass site and marked with a metal stake. We explored whether the genotype of BA found in the carcass had any effect on the initial concentrations or persistence in soil but found no significant effects, therefore the genetic diversity of the pathogen was not considered further.

Soil characteristics and effects on BA persistence

Additional soil samples (four 10cm cores, pooled by site) were collected near carcass sites to test if soil characteristics altered *Bacillus anthracis* (BA) persistence over time, measuring soil pH, electrical conductivity, organic matter, P, K, Ca, Mg, and Na. These cores were collected in each direction 9m from the marking stake at the center of the site. This is because carcasses can significantly alter soils[1] and we were interested in local soil conditions without the effect of the carcass, and to protect the health of laboratory workers conducting soil analyses. One sample was collected per site in 2012/2013 and a second in 2014. All soils were analyzed at the Ministry of Agriculture, Water and Forestry Laboratory in Windhoek, Namibia. When controlling for differences in mass and site age, no significant differences in CFU/g could be found in relation to any of the soil characteristics (all $p>0.1$; soil characteristics summarized in Table S3). These patterns persisted for the length of the study, suggesting that the soils throughout the study area are quite similar, with no detectable effects of soil properties on persistence.

qPCR protocol for carcass surface soil samples

The PCR was augmented to alleviate inhibition from the soil samples by increasing the primer concentration from 0.6µM to 1.2µM, adding non-acetylated BSA to a final 1mg/ml, and diluting the extracted DNA by 1:10. TaqMan® Exogenous Internal Positive Control (Life Technologies) was run together with plcR taqMAMA to control for inhibition and variability between reactions. Absolute quantification was performed by making a standard 1:10 dilution series using BA DNA extracted from pure culture. The BA DNA was first quantified using a Qubit® Fluorometer (Life Technologies), adjusted to1ng/µL then a serial dilution was made spanning the range 1ng to 10fg. All DNAs including the standard dilution from soil samples were run in triplicate on a LightCycler® 96 (Roche) express qPCR super mix. Samples were quantified using LightCycler® Roche 96 software 1.1, taking the average of the triplicates and then adjusting to equivalent genomes/g soil dry matter.

Estimating a standard curve for water sediment Ct-values

Two gram of a mixture of dry soil from 4 different gravel pits was placed onto gauze in a 9 cm glass petri dish and 50 µl of a spore suspension of Sterne strain 34F2, containing $2x10^2$, $2x10^3$, $2x10^4$ or $2x10^5$ spores was added to the soil layer. After complete drying, each 2g sample was re-suspended in 5 ml of water and completely used to cover 4 TSPBA plates. After overnight incubation at 37 °C the bacterial lawns were scraped off with 1 ml per plate, and then treated as described above. The Ct-values obtained from this were connected to the spore counts through a Poisson regression and used as a standard curve to estimate spore counts from Ct-values for the waterhole data.

Four PCR trials were done from each spiked sample, and the relationship between Ct-values and the (known) number of BA-spores per gram of sediment was assessed by a generalized additive mixed model (GAMM) with replicate as the random variable. BA negative samples were treated as Ct>40. The relationship was found to be close, with Ct-values explaining 87% of the deviance in BA concentrations. The resulting model was used as a standard curve transferring Ct-values from waterhole sediment samples to estimates of BA CFU/g (Table S4 and inset figure). The predicted BA counts were corrected for percent dry matter in the samples to be comparable to carcass soil samples given in BA per gram dry soil. The estimated BA sediment densities were then tested with multiple regressions versus candidate explanatory variables (waterhole type, time of year, time since sampling initiated, whether sediments were wet or dry). The main effect turned out to be type of waterhole, with seasonal water sources having significantly more BA than the perennial sources (even when controlling for time and resampling by mixed models.

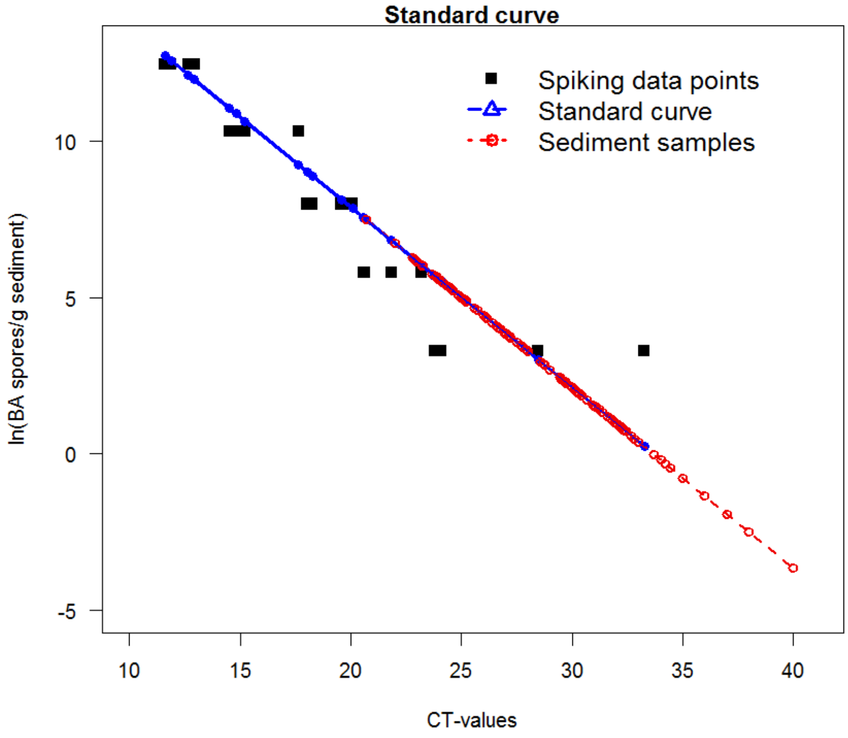

Inset figure: The standard curve relating Ct-values to spiking experiment data points. The resulting estimates of BA concentrations from sediment sample Ct-values are shown in red.

BA on grasses data processing

We standardized the measures of BA concentrations on the above-ground component of grasses for the exposure model, since sampling protocols differed slightly between samples from 2012-2013 collected by Turner et al.[1] and additional samples collected as part of this study. In Turner et al.[1], the above-ground component was kept as a single sample, whereas in this study the above-ground component was divided into "tops" and "bases." For each plant for which separate "top" and "base" samples existed, the concentrations of BA on the "top" and "base" were averaged and weighted by the sample mass to get a concentration for the combined above-ground component. Roots, plant–associated soil samples, and surface soil samples were kept as separate observation sets from the above-ground grass data.

Supplementary Results

Quantifying BA in carcass site soils comparing culture to qPCR

For surface soil samples comparing culture to qPCR (N=110, includes replicate samples), 96 were positive and three were negative for BA from both methods, an agreement of 90%). Where the two methods did not agree, six samples were PCR positive/culture negative and five were culture positive/PCR negative. As evident from Figure 3, the samples where the two methods disagreed were not only at low BA concentrations, and thus may represent variation in the sub-samples of soils used for analysis. We found a strong positive relationship between the estimates of BA concentrations in soils from culture and from qPCR (t=7.81, p<0.0001, R^2=0.504, N=62 sites), although the estimates diverged from a 1:1 line as concentrations increased beyond approximately 10^4 CFU/g (Figure 3). This divergence may be due to lower efficiency in the DNA extraction or PCR kinetics and requires further study. The DNA extraction efficiency from the spiking experiment was 7.8% (SD 5.7%) at a concentration of 3.4 x 10^6 spores. We found a strong correlation between replicate qPCR estimates at sites with a slope that did not differ from the 1:1 line (R^2=0.517, N=48 sites).

Effect of grazing different portions of the above-ground biomass on exposure

The grazing exposure models in the main text explore the effect of consuming only the above-ground grass component (equation 2) or this component coupled with a proportion of root and soil ingestion (equation 3). Given the result that grass bases held significantly higher concentrations of BA than grass tops (p=0.0004), we explored what effect ingesting different parts of the above-ground component may have on BA exposure. However, we did not include this as a separate exposure model because i) there was significantly less data collected where the above-ground components were differentiated, and ii) we do not know how to describe a community of individuals foraging over time at different heights in a heterogeneous grazing mosaic that would capture key properties relevant to disease transmission. Thus, we explore what effect consuming different portions of the above ground component might have on our model results.

We assessed the effect of disproportionally ingesting tops versus bases on individual grass i by calculating an exposure rate, S, signifying the effect of selectively eating grass tops by a proportion $P_{top.}$

$$S = \frac{\frac{C_{top,i}\,W_{top,i}P_{top}+C_{base,i}\,W_{base,i}(1-P_{top})}{W_{top,i}P_{top}+W_{base,i}(1-P_{top})}}{\frac{C_{top,i}\,W_{top,i}+C_{base,i}\,W_{base,i}}{W_{top,i}+W_{base,i}}} \qquad \text{(eq. S1)}$$

(P_{top} ranges from 0 to 1, with 0 meaning no intake of grass tops, only of bases (i.e. grazing at the site after others had consumed the tops), 0.5 means a proportional intake (i.e. simply eating the whole above-ground component), and 1 meaning no intake of plant bases, grazing solely on tops. A range of *S* was obtained for each value of P_{top} by bootstrapping the observed CFU/g dry matter with Poisson resampling so that $C_{top,i}$~Poisson($CFUg_{top,i}$) and $C_{base,i}$~Poisson($CFUg_{base,i}$) with corresponding sample weights, *W*. The resulting distribution of *S* (inset figure) shows the estimated effect. While not extremely strong, it does point out that animals grazing at the base of the plant, possibly because the top was previously eaten, suffer greater exposure to BA. If animals manage to disproportionally eat grass tops without ingesting grass bases, this might reduce their exposure to BA by up to two orders of magnitude. This effect could magnify seasonal differences in exposure, when balancing the role of rainfall on grass regeneration, with numbers of grazers on the landscape to consume available grass biomass.

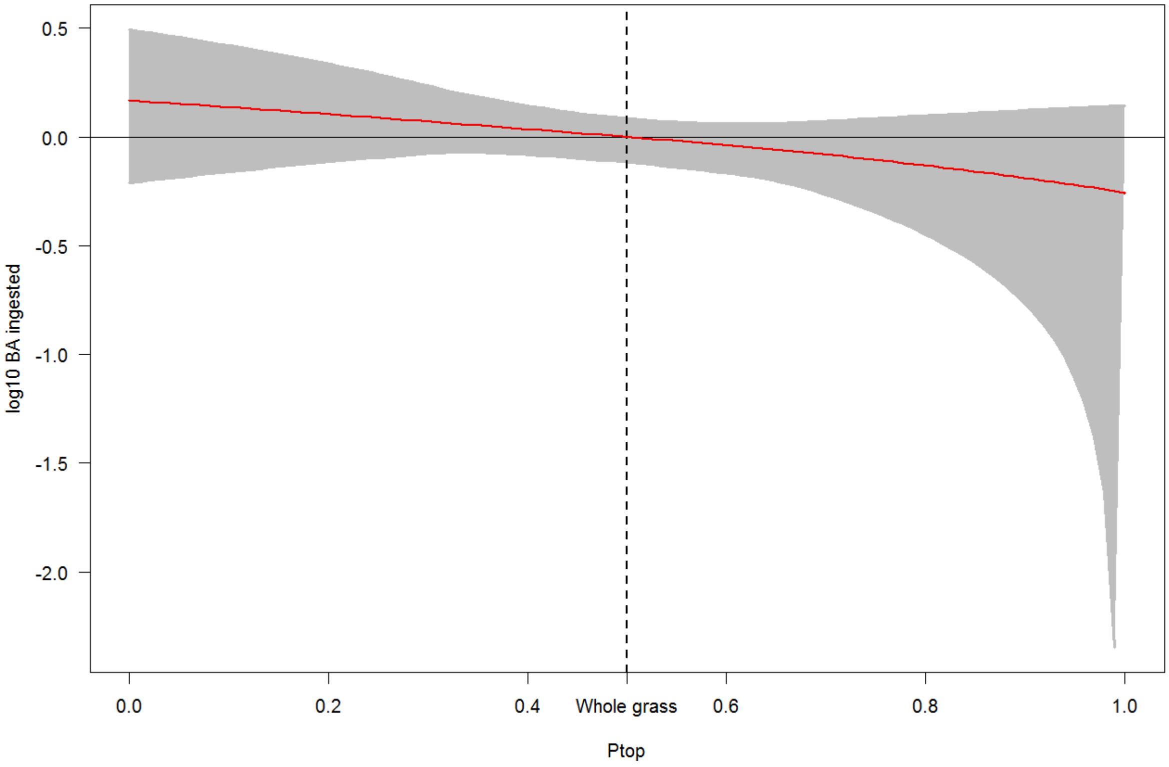

Inset figure. The effect of changing the ratio of grass tops to grass bases on the exposure to BA. On the P_{top} axis, “whole grass” means ingesting the entirety of the above-ground component of the grass, 0 means only ingesting bases, 1 means only ingesting tops. The 90 percentile (gray area) and mean (red line) of *S* (see equation above) following 1E5 Poisson-redraws from observed samples for each value of P_{top} are shown on a $\log_{10}$ scale.

Table S1. Experimentally assessed oral lethal doses of *Bacillus anthracis* spores. Limited data exist from studies of lethal doses for BA in different animal species. What data are available come primarily from experimental vaccine trials or trials evaluating the course of infection, where the pathogen is injected parenterally to control for entry point, exposure amount and time since exposure. This is unfortunate for understanding the transmission of the disease in natural systems, since injection is not among the natural routes of infection. The inhalational infection route has been a focus of recent research efforts[2, 3, 4] due to concerns over bioterrorism and the risk of inhalational anthrax to humans. However, since ingestion is thought to be most important for non-human animals, we restrict this table to data on the oral infection route.

Species	Spores administered	How administered	Doses that were lethal to naive animals[a]	Number of naive dead/live after trial	Reference
cattle	$6x10^8$	inside gelatin capsule	non-lethal	0/1	5
cattle	10^9	inside feed pellet	10^9	4/1 to 5/0*	5
cattle	10^7	inside feed pellet	10^7	2/1	5
cattle	10^8	inside feed pellet	10^8	2/1 to 3/0*	5
cattle	$1.5x10^8$	inside feed pellet	$1.5x10^8$	2/1 to 3/0*	5
cattle, sheep, horses	$5x10^8$	gastric tube or in oats and bran	5x10^8 a "practical lethal dose"	unknown	6 (describes studies by British Biological Department, Porton in 1940s)
guinea pigs/rabbits	10^8	feeding	"repeatedly failed to induce infection"	unknown	7
guinea pigs/rabbits	$1.2x10^6$	spores in milk to newborn animals	non-lethal	unknown	8
impala	unknown	unknown	$1x10^6$ - $6.5x10^7$	unknown	unpublished study described in 9
mice	10^5†	injected orally with a feeding needle after fasting 4 hrs	non-lethal	0/10	10

mice	10^7†	injected orally with a feeding needle after fasting 4 hrs	10^7	2/8	10
mice	10^9†	injected orally with a feeding needle after fasting 4 hrs	10^9	8/2	10
mice	$5x10^8$ twice	in drinking water in two subsequent days	non-lethal	0/10	11 (translation 1962 by US Army Biological Laboratories)
pigs	$1.6x10^7$ - $1.1x10^{10}$	in feed with grit; 1, 2 or 3 exposures	$1.6x10^7$ and $7.8x10^7$	2/48	12
pigs	10^6	not specified	non-lethal	0/15	5
pigs	innumerable‡	ingested entire guinea pig carcass recently dead from anthrax	non-lethal	0/8	5
pigs	innumerable‡	ingested 2 entire guinea pig carcasses recently dead from anthrax (1/day for 2 days)	non-lethal	0/2	5

[a]numbers shown are for naïve animals only, vaccinated animals in vaccine studies are excluded

*surviving individual died on re-challenge

†challenges used the Sterne strain and mice are highly susceptible to this strain

‡these were probably vegetative cells, not spores, so maybe not surprising did not induce an infection. However, did cause the most severe symptoms so the guinea pig method was used for subsequent vaccine trials in the study. Pigs are known to be particularly resistant to *B. anthracis.*

Table S2. Concentrations of *Bacillus anthracis* found naturally in the environment. Samples of soil, air, water, feces and vegetation were collected in areas associated or not with a known anthrax case. We do not report concentrations for samples evaluated at managed sites (e.g., where carcass burnt or buried) or human-altered environments (e.g., near tanneries). Several additional studies reported the presence/absence of *B. anthracis* in environmental samples; however this table is restricted to studies that quantified concentrations. *Bacillus anthracis* concentrations were reported in CFU (colony forming units) or spores per unit of sample material. Sample processing methods varied among studies, and differences are highlighted following the categories of Silvestri et al.[13] Samples were cultured on either BA (blood agar) or PLET (Polymyxin B, Lysozyme, EDTA, Thallous acetate agar); confirmation subcultures included tests for gamma phage and penicillin sensitivities and lack of hemolysis. Study locations included Etosha National Park (ENP), Namibia; Wood Bison National Park (WBNP), Canada; and Luangwa Valley (LV), Zambia.

Associated with anthrax site?	Sample type	Sample source	Positive samples /total	Range of concentrations	Sample processing method	Identification method	Location	Reference
yes	air	filtered air 6-18m downwind from 3 anthrax carcasses (w/ and w/o soil disturbance)	14/30	0 - 0.021 CFU/L	cyclone sampler with aqeous carrier, heat	culture (BA and PLET)	ENP, Namibia	14
yes	air	filtered air 6-18m downwind from 3 anthrax carcasses (w/ and w/o soil disturbance)	3/14	0- 0.0071 CFU/L	gelatin air filters, filter applied directly to agar plate, dissolved	culture (PLET)	ENP, Namibia	14
yes	feces	vulture near anthrax carcass	9/18	2-174 CFU/g	aqueous carrier, agitation, settling, heat	culture (PLET), some subculture for confirmation	ENP, Namibia	15
yes	feces	hyena near anthrax carcass	3/5	2-20,000 CFU/g	aqueous carrier, agitation, settling, heat	culture (PLET), some subculture for confirmation	ENP, Namibia	15

yes	feces	jackal near anthrax carcass	18/25	2-4,480 CFU/g	aqueous carrier, agitation, settling, heat	culture (PLET), some subculture for confirmation	ENP, Namibia	15
yes	grass	grass roots at anthrax positive carcass sites (26 sites, 3 grasses/site)	56/78	0-330,200,000 CFU/g	aqueous carrier, vortexing, low and high speed centrifugation	culture (PLET) some subculture for confirmation	ENP, Namibia	1
yes	grass	above ground grass component at anthrax positive carcass sites (26 sites, three grasses/site)	45/78	0-50,800,000 CFU/g	aqueous carrier, vortexing, low and high speed centrifugation	culture (PLET) some subculture for confirmation	ENP, Namibia	1
yes	soil	at and near anthrax carcass sites (sub-sampled 7 sites)	160/250	0-1,000 spores/g	aqueous carrier, agitation, high specific gravity separation, centrifugation, (heat or ethanol?)	culture (PLET), some subculture for confirmation tests, PCR	WBNP, Canada	16
yes	soil	soil surrounding grasses at anthrax positive carcass sites (26 sites, 3 samples/site)	66/78	0-11,500,000 CFU/g	aqueous carrier, vortexing, low and high speed centrifugation	culture (PLET) some subculture for confirmation	ENP, Namibia	1
yes	soil	anthrax carcass sites (one sample/site)	106/106	1->1,000,000 spores/g	aqueous carrier, agitation, settling, heat	culture (PLET), some subculture for confirmation tests	ENP, Namibia	15
yes	soil	known or suspected anthrax carcass sites	1/8	0-180,000 spores/g	aqueous carrier, agitation, settling, heat	culture (BA and PLET), subculture for confirmation	LV, Zambia	17
yes	water	during outbreak in elephants	19/73	1-22spores/ml	heat	culture (PLET), some subculture for confirmation	ENP, Namibia	15

no	soil	gravel pits, pans, springs, boreholes	7/230	4-80 spores/g	aqueous carrier, agitation, settling, heat	culture (PLET), some subculture for confirmation	ENP, Namibia	15
no	water	gravel pits, pans, springs, boreholes	3/92	0-1 spores/ml	heat	culture (PLET), some subculture for confirmation	ENP, Namibia	15
no	water	gravel pits, pans, springs, boreholes	1/12	0-100 CFU/ml	heat	culture (PLET), some subculture for confirmation	ENP, Namibia	18

Table S3. Average soil characteristics in the study area. Soil samples were collected outside of marked carcass sites on two occasions, to record the background soil without the influence of the carcass. **S**ample 1 was from 2012-2013 for N=38 sites and sample 2 was from 2014 for N=41 sites.

		**Sample 1**		**Sample 2**	
**Soil characteristics**	**Units**	**Mean**	**SD**	**Mean**	**SD**
pH		8.6	0.2	8.6	0.1
Electrical conductivity	µS/cm	145.2	40.5	245.2	91.6
Organic matter	%	2.0	0.5	2.0	0.4
Phosphorus	ppm	2.8	2.1	6.5	3.2
Potassium	ppm	424.4	234.6	1611.7	637.0
Calcium	ppm	4196.0	333.8	3700.2	1110.7
Magnesium	ppm	376.2	131.3	559.3	264.4
Sodium	ppm	144.5	334.4	172.0	243.5
Sand	%	48.3	11.6	48.3	8.1
Silt	%	42.7	10.4	44.0	7.7
Clay	%	9.0	3.7	7.7	2.8

Table S4. Water sources sampled for *Bacillus anthracis* in Etosha National Park, Namibia. The number of unique sampling events in time, the total number of samples collected, the numbers positive for *B. anthracis*, and sample prevalence is identified for each location. *Bacillus anthracis* concentrations were quantified by qPCR and linked to spore concentrations (in CFU/g) by spiking known quantities of spores to soil samples (SI Methods).

**Water source**	**Sites investigated**	**Sampling dates (range by month/year)**	**N sampling events**	**N samples positive/total**	**Prevalence**	**Mean CFU/g (range)**
seasonal	3km E of Okaukuejo	11/2009-4/2013	16	15/20	0.75	72 (0-1946)
	Adamax	11/2009-3/2013	13	30/38	0.79	238 (<1-845)
	Airfield Okaukuejo	11/2009-3/2013	16	19/27	0.70	71 (0-845)
	Grünewald	11/2009-4/2013	14	8/14	0.57	6 (0-100)
	Leeubron	2/2010-3/2011	9	7/9	0.78	8 (0-26)
	Natco	2/2010-3/2011	8	8/8	1.00	123 (<1-845)
natural spring	Andoni	4/2010-5/2010	2	1/4	0.25	67 (0-266)
	Chudop	11/2009-3/2011	10	5/10	0.50	12 (0-47)
	Groot Okevi	11/2009-3/2011	9	1/9	0.11	0.5 (0-5)
	Kameeldoring	5/2010-6/2010	2	0/10	0.00	0 (0)
	Klein Namutoni	5/2010-3/2011	3	0/5	0.00	0 (0)
	Klein Okevi	11/2009-11/2010	8	1/8	0.13	3 (0-26)
	Mushara	4/2010	1	0/2	0.00	0 (0)
	Rietfontein	11/2009-3/2011	4	1/15	0.07	0.3 (0-5)
	Salvadora	11/2009-3/2011	9	6/3	0.67	5 (0-26)
borehole	Arendsnes	11/2009-3/2010	2	1/3	0.33	0.2 (0-<1)
	Aus	11/2009-4/2010	2	0/4	0.00	0 (0)
	Duiwelsvuur	11/2009-4/2010	2	0/4	0.00	0 (0)
	Gemsbokvlakte	5/2010-1/2011	6	7/7	1.00	64 (3-84)
	Olifantsbad	11/2009-4/2010	2	0/4	0.00	0 (0)
	Ombika	2/2010-9/2010	6	2/6	0.33	0.5 (0-3)
	Ozonjuitji m'bari	11/2009-3/2010	2	2/5	0.40	0.5 (0-1)
	Sonderkop	11/2009-3/2011	10	7/9	0.78	3 (0-26)

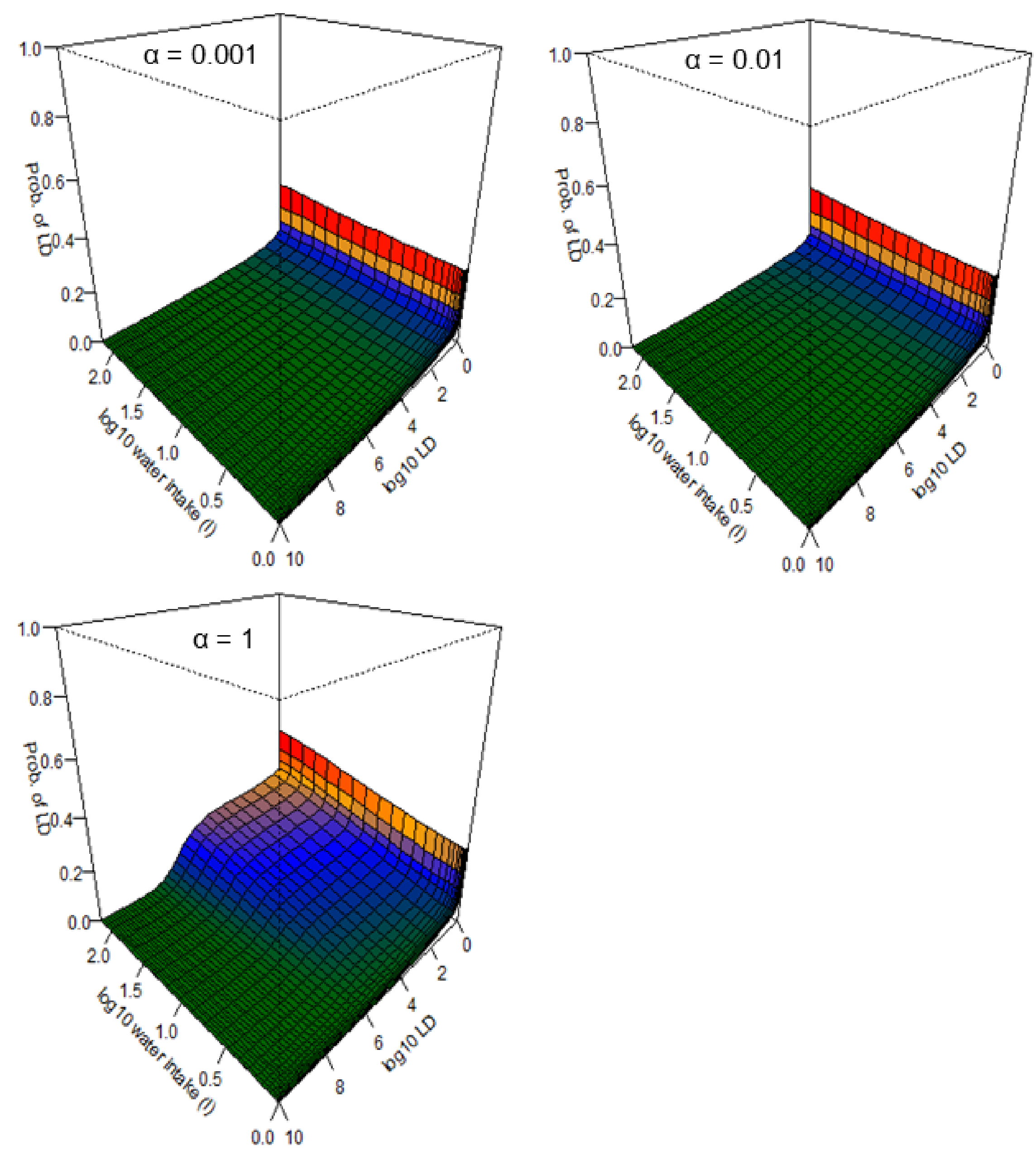

Figure S1. The probability of ingesting a lethal dose (LD) of *Bacillus anthracis* from drinking at boreholes. BA concentrations were measured in sediments below the water, and how much *B. anthracis* may be found in water is unknown, but can be assumed to be much lower than in sediments. Therefore we show a range of possible exposures, where the amount in water is $1/1000^{th}$ what is found in sediments (α=0.001), where the amount in the water is $1/100^{th}$ what is found in sediments (α=0.01), and an unrealistic, worst-case scenario where the amount of *B. anthracis* in the water is the same (per unit mass) as the amount in the sediments below (α=1) (see equation 4). Results for other water sources are shown in Figure 6 and the axes and colors are consistent between the two figures.

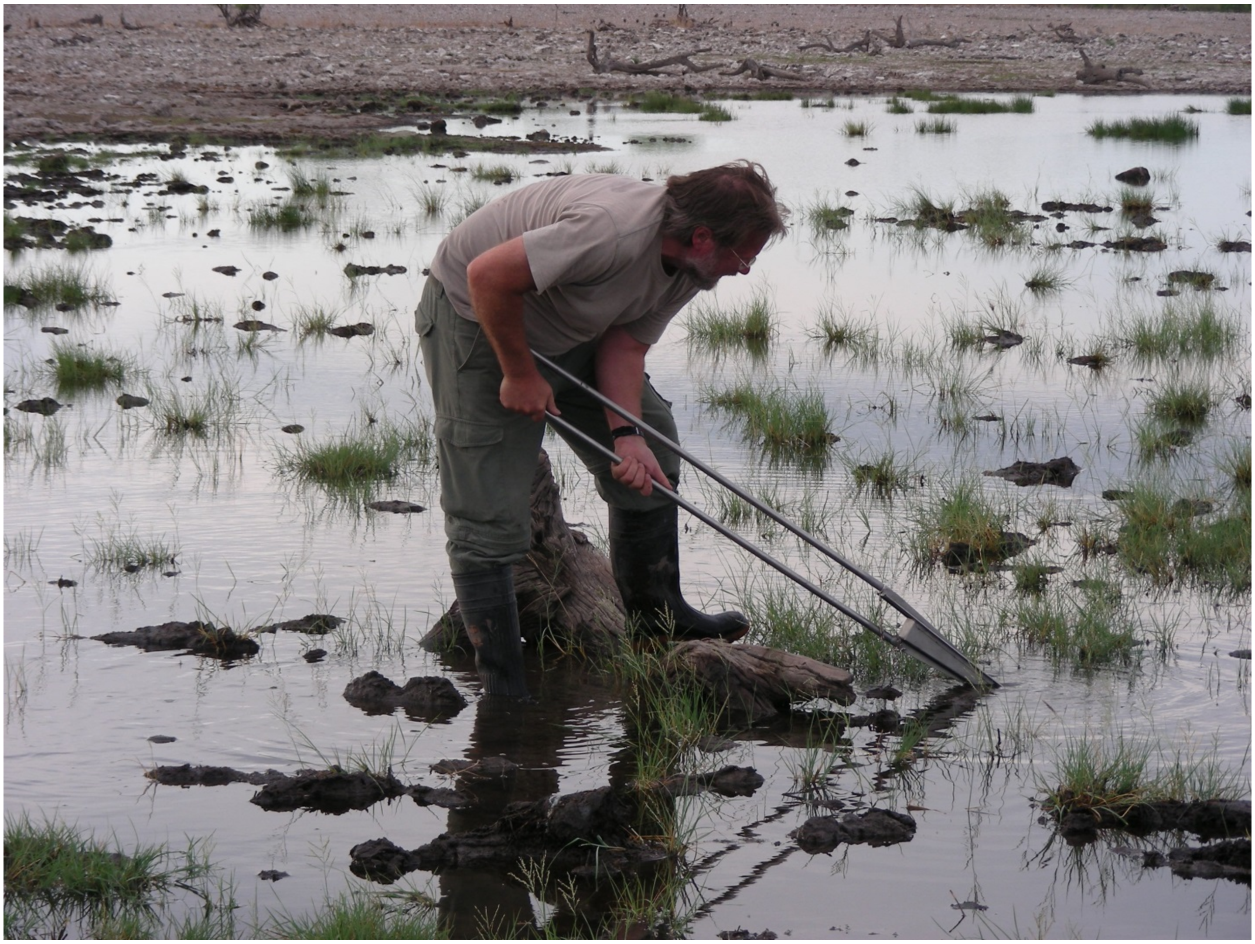

Figure S2. Sampling protocol for sediments below water sources. When a sampling location was under water, sediment samples were collected using a shovel-like device, consisting of a 40x8cm shovel with a movable lid, both fitted to steel rods of 3m length. When using the shovel, the 50-100g sample was collected by randomly subsampling the shovel’s contents.

References

1. Turner, W. C. *et al.* Fatal attraction: vegetation responses to nutrient inputs attract foraging herbivores to infectious anthrax carcass sites. *Proc. R. Soc. Lond. [Biol]* **281**, 20141785 (2014).

2. Gutting, B.W. *et al.* Modeling low-dose mortality and disease incubation period of inhalational anthrax in the rabbit. *J. Theo. Biol.* **329**, 20-31 (2013).

3. Haas, C.N. On the Risk of Mortality to Primates Exposed to Anthrax Spores. *Risk Analysis* **22**, 189-193 (2002).

4. Bartrand, T.A., Weir, M.H. & Haas, C.N. Dose-response models for inhalation of *Bacillus anthracis* spores: interspecies comparisons. *Risk Anal.* **28**, 1115-1124 (2008).

5. Schlingman, A.S., Devlin, H.B., Wright, G.G., Maine, R.J. & Manning, M.C. Immunizing activity of alum-precipitated protective antigen of *Bacillus anthracis* in cattle, sheep and swine. *Am. J. Vet. Res.* **17**, 256-261 (1956).

6. Carter, G.B. & Pearson, G.S. British biological warfare and biological defence, 1925-45 in *Biological and toxin weapons: research, developments and use from the Middle Ages to 1945*. (eds Geissler, E. & van Courtland Moon, J.E.) pp 168-189 (SIPRI No. 18, Oxford University Press Oxford, UK, 1999)

7. Druett, H.A., Henderson, D.W., Packman, L. & Peacock, S. Studies on respiratory infection. I. The influence of particle size on respiratory infection with anthrax spores. *J. Hyg-Cambridge* **51**, 359-371 (1953).

8. Sanarelli, G. The pathogenesis of "internal" or "spontaneous" anthrax. *J. Comp. Pathol. Ther.* **38**, 135-141 (1925).

9. de Vos, V. The ecology of anthrax in the Kruger National Park, South Africa. *Salisbury Med. Bull.* **68S**, 19-23 (1990).

10. Lightfoot, Y.L. *et al.* Colonic immune suppression, barrier dysfunction, and dysbiosis by gastrointestinal *Bacillus anthracis* infection. *Plos One* **9**, e100532 (2014).

11. Velu, H. & Bellocq, B. Pathogenicity of anthrax spores in white mice. *Comptes Rendus de la Société de Biologie* **134**, 1352-1353 (1941).

12. Redmond, C., Hall, G.A., Turnbul, P.C.B. & Gillgan, J.S. Experimentally assessed public health risks associated with pigs from farms experiencing anthrax. *Vet. Rec.* **141**, 244-247 (1997).

13. Silvestri, E.E., Perkins, S.D., Feldhake, D., Nichols, T. & Schaefer, F.W., III. Recent literature review of soil processing methods for recovery of *Bacillus anthracis* spores. *Ann. Microbiol.* 1-12 (2014).

14. Turnbull, P.C.B., Lindeque, P.M., Le Roux, J., Bennett, A.M. & Parks, S.R. Airborne movement of anthrax spores from carcass sites in the Etosha National Park, Namibia. *J. Appl. Microbiol.* **84**, 667-676 (1998).

15. Lindeque, P.M. & Turnbull, P.C.B. Ecology and epidemiology of anthrax in the Etosha National Park, Namibia. *Onderstepoort J. Vet. Res.* **61**, 71-83 (1994).

16. Dragon, D.C., Bader, D.E., Mitchell, J. & Woollen, N. Natural dissemination of *Bacillus anthracis* spores in northern Canada. *Appl. Environ. Microbiol.* **71**, 1610-1615 (2005).

17. Turnbull, P.C.B. *et al.* Anthrax in wildlife in the Luangwa Valley, Zambia. *Vet. Rec.* **128**, 399-403 (1991).

18. Turnbull, P.C.B. *et al.* Further progress in understanding Anthrax in the Etosha National Park. *Madoqua* **16**, 93-104 (1989).
